# Prolactin promotes proliferation of germinal center B cells, formation of plasma cells, and elevated levels of IgG3 anti-dsDNA autoantibodies

**DOI:** 10.3389/fimmu.2022.1017115

**Published:** 2022-10-25

**Authors:** Ricardo Carreón-Talavera, Paola Santana-Sánchez, Ezequiel Moisés Fuentes-Pananá, María Victoria Legorreta-Haquet, Luis Chávez-Sánchez, Patricia Sofia Gorocica-Rosete, Adriana Karina Chávez-Rueda

**Affiliations:** ^1^ UIM en Inmunología, Hospital de Pediatría, CMN Siglo XXI, Instituto Mexicano del Seguro Social, México City, Mexico; ^2^ Unidad de Investigación en Virología y Cáncer, Hospital Infantil de Mexico Federico Gómez, México City, Mexico; ^3^ Departamento de Investigación en Bioquímica, Instituto Nacional de Enfermedades Respiratorias “Ismael Cosió Villegas”, México City, Mexico

**Keywords:** prolactin, systemic lupus erythematosus, B cells, germinal center, STAT1

## Abstract

Systemic lupus erythematosus (SLE) mainly affects females at reproductive age, which has been associated with hormones, such as prolactin (PRL). Different studies suggest that PRL exacerbates the clinical manifestations of SLE both in patients and in mouse models (e.g., the MRL/lpr strain), increasing the production of autoantibodies, which can be deposited as immune complexes and trigger inflammation and damage to different tissues. The objective of this work was to explore the potential mechanisms by which PRL increases the concentration of self-reactive antibodies in the MRL/lpr SLE model. To this end, we determined the role of PRL on the activation and proliferation of germinal center B cells (B-GCs) and their differentiation into antibody-secreting cells (ASCs). We show that the absolute number and percentage of B-GCs were significantly increased by PRL *in vivo* or upon *in vitro* treatment with anti-IgM and anti-CD40 antibodies and PRL. The augmented B-GC numbers correlated with enhanced proliferation, but we did not observe enhanced expression of CD80 and CD86 activation markers or the BCL6 transcription factor, arguing against a more effective differentiation. Nevertheless, we observed enhanced phosphorylation of STAT1, secretion of IL-6, expression of IRF4, numbers of ASCs, and levels of IgG3 antibodies directed against dsDNA. Altogether, these results support the hypothesis that a PRL-mediated expansion of B-GCs yields more self-reactive ASCs, potentially explaining the pathogenic immune complexes that steadily lead to tissue damage during SLE.

## Introduction

The germinal centers (GCs) are located in the follicular zone of the secondary lymphoid organs. In the GCs, the isotype switching and somatic hypermutation are carried out, resulting in the terminal differentiation of memory B cells and high-affinity antibody-secreting cells (ASC), both important for protection against pathogens (1). GC deregulation contributes to the progression of autoimmune diseases. In mouse and human models with autoimmunity, the appearance of spontaneous GC has been identified, correlating with the presence of autoantibodies (2, 3). In addition, autoreactive clones (9G4-B cells) that evade GC checkpoints have been reported in systemic lupus erythematosus (SLE) (4).

SLE is a multifactorial, chronic, autoimmune disease characterized by an exacerbated immune response, mainly of B-cells, which explains the presence of autoantibodies directed against various molecules of the nucleus, such as DNA, RNA, Ro, La, and histones. These autoantibodies form immune complexes that often leave the circulation and are deposited in the kidney, skin, and brain, among other tissues, causing inflammation and tissue damage (5, 6). SLE predominantly affects women rather than men in a 9:1 ratio, especially young women of reproductive age (7, 8). The gender dimorphism of SLE is usually attributed to the fact that alterations in the immune-neuroendocrine system contribute to the development of autoimmunity, and therefore it is suggested that hormones, such as prolactin (PRL), modulate the immune response influencing the development of SLE (9, 10). Indeed, 15-33% of lupus patients have elevated levels of serum PRL that correlate with disease activity (11–13), and individuals with hyperprolactinemia have an increased prevalence of autoantibodies (especially anti-dsDNA) (14).

In model mice that develop SLE, it has been reported that elevated levels of PRL exacerbate the disease, correlating with the production of autoantibodies and elevated markers of kidney damage, such as increased proteinuria (15–17). PRL has different biological functions that depend on the interaction with its receptor, for which different isoforms have been described. In mice, four isoforms have been identified, one long and three short, with the long isoform signaling through JAK-STAT, MAPK, and PI3K-AKT and the short isoform only signaling through MAPK and PI3K-AKT (18, 19).

Our group has reported that the different stages of maturation of B cells in bone marrow (proB, preB, and immature) (15), and in the spleen (transitional, follicular [FO] and marginal zone [MZ]) express the PRL receptor (16), both in strains that do not develop SLE (C57BL/6) and in models affected by the disease (MRL/lpr). In addition, we identified that PRL influences the B-cell maturation process by rescuing immature autoreactive B cells from apoptosis (20, 21). However, the mechanism by which PRL may favor autoantibody production is unclear.

In this study, we assessed *in vivo* and *in vitro* the possible mechanisms by which PRL could influence the GC reaction and the differentiation of GC B cells (B-GCs) to favor the production of autoantibodies. We addressed the role of PRL in the activation, differentiation, and proliferation of B-GCs and found that PRL increased the absolute number and percentage of these cells in the MRL/lpr strain. The augmented B-GC percentage correlated with enhanced proliferation and enhanced levels of anti-dsDNA IgG3 antibodies and IL-6. We also observed a mostly exclusive expression of the long isoform of the PRL receptor and signaling through activated STAT1 and AKT in B-GCs. The augmented B-GCs favored the formation of more ASCs, probably explaining the levels of self-reactive antibodies.

## Materials and methods

### Mice

The Animal Care Committee of the Instituto Nacional de Enfermedades Respiratorias (INER) “Ismael Cosío Villegas” and the Hospital de Pediatría, CMN Siglo XXI, IMSS, approved the experiments performed in this study (R-2021-3603-029 and R-2016-785-050). Mice were worked according to the NIH Guide for the Care, the guidelines established in Mexico (NOM-062-ZOO-1999) and Use of Laboratory Animals. The C57BL/6 mice were acquired from Instituto Nacional de Enfermedades Respiratorias (INER) “Ismael Cosío Villegas” (Mexico City, Mexico), and the MRL/lpr (MRL/MpJFASlpr) and MRL (MRL/MpJ) mice were acquired from the Jackson Laboratory (Bar Harbor, Maine, USA). All mice were provided sterile food and water *ad libitum*, and sheltered in pathogen-free facilities.

### Prolactin hormone

Highly purified recombinant mouse PRL (AFP-405C) was obtained from Dr A.F. Parlow, at the National Hormone and Peptide Program, Harborn UCLA Medical Center, CA, USA.

### Antibodies

For cell culture, the following antibodies were used: anti-CD40 (rat IgG2a clone FGK45.5) from Miltenyi Biotec (Bergisch Gladbach, Germany) and anti-IgM F(ab´)_2_ goat polyclonal antibody from Jackson ImmunoResearch (West Grove, PA, USA). For cell staining, the following antibodies were used: anti-CD19 FITC (clone 1D3/CD19), anti-GL-7 Pacific Blue (clone GL7), anti-Ki-67 Alexa 488 (clone 16A8), anti-pSTAT1 PE (clone A15158B), anti-pSTAT3 PE (clone 13A3-1), anti-pERK1/2 PE (clone 6B8B69), and anti-IL-6 PE (clone MP5-20F3) from BioLegend (San Diego, CA, USA), anti-CD19 PerCP-Cy5.5 (clone 1D3), and anti-CD21 APC (clone 7G6) from BD Biosciences (Mountain View, CA, USA), anti-CD138 PE (clone 30506), anti-CD23 PE-cyanine7 (clone B3B4) from Invitrogen (Carlsbad, CA, USA), anti-pSTAT5 PE (clone SRBCZX), anti-CD80 PE (clone 16-10A1), anti-CD86 PE (clone GL1), anti-BCL6 PE (clone BCL-DWN), and anti-IRF4 PE-Cy7 (clone 3E4) from eBioscience (San Diego, CA, USA), and anti-pAKT PE (clone REA359) from Miltenyi Biotec.

### Induction of hyperprolactinemia and evaluation of SLE manifestations

Eight-week-old C57BL/6 and MRL/lpr female mice were treated subcutaneously with (i) metoclopramide (200 µg, Sigma−Aldrich, St. Louis, MO, USA) in 100 µL of PBS with five doses per week for six weeks. The control group received (ii) 100 μL of PBS or (iii) no treatment during the same period. Using reactive strips for urinalysis (Mission, San Diego, CA, USA) were used to measure the urinary protein levels

### Analysis of antibodies in serum

Serum samples were taken from mice at the beginning (eight weeks) and end (15 weeks) of the experiments and were stored at -35°C until anti-dsDNA (15), and anti-Histone (22) antibodies were analyzed by ELISA as follows. A MaxiSorp plate (Nunc, Rochester, NY, USA) from 96-well was coated with 2.5 μg/ml calf thymus dsDNA (Sigma Aldrich, St Louis, MO, USA) or 10 μg/ml calf thymus histone (Roche Diagnostic, Mannheim, Germany) in 100 μl of bicarbonate buffer overnight at 4°C. Bovine serum albumin (BSA, Invitrogen, Carlsbad CA, USA) at 2% was used to block the plate. The plate was incubated for one hour at 37°C with serum (1:50) or the anti-dsDNA antibody standard (clone 16-13, Chemicon International, Billerica MA, USA) or was incubated for two hours at room temperature with serum (1:150) for anti-histone antibody. The plate was washed and incubated with rabbit anti-mouse IgM, IgG, IgG1, or IgG2a conjugated to alkaline phosphatase (AP, Zymed Laboratories, San Francisco CA, USA) or anti IgG2b or IgG3 conjugated to peroxidase (HRP), after which substrate was added. (5-bromo-4-chloro-3- indolyl phosphate; Sigma−Aldrich, St Louis MO, USA) for AP or HRP substrate (3.3’,5,5’ tetramethylbenzidine TMB Sigma−Aldrich), respectively. The O.D. was read at 405 or 450 nm using a Dynatech MR5000 ELISA reader.

### B-cell purification

B cells were purified by negative selection, for which eight-week-old female mice were euthanized. Spleen cells were collected with cold RPMI (Gibco, Grand Island, NY, USA). The erythrocytes were eliminated using lysis buffer (Sigma−Aldrich), and the splenocytes were incubated with anti-CD43 (Ly-48) antibody conjugated with magnetic beads (Miltenyi Biotec). The cells went through a MACS LD separation column (Miltenyi Biotec). A >96% purity was determined through flow cytometry.

### Cell sorting

Purified B cells from spleen from eight-week-old female (for FO cell) or from 15-week-old female (for B-GCs) MRL/lpr mice were incubated with Ghost Red marker from Tonbo Biosciences (San Diego, CA, USA) and with the following antibodies specific for CD19, CD21, and CD23 (for FO cells) or CD19 and GL7 (for B-GC) in FACS buffer (PBS-BSA 0.5%) for 20 minutes at 4°C. The cells were washed, and FO or B-GC cells were sorted depending on the expression of CD19^+^CD21^int^CD23^+^ or CD19+GL7+, using a FACSAria sorter (BD Bioscience). The purity of the sorted cells ranged from 95% to 98%.

### *In vitro* differentiation of germinal center B cells and antibody-secreting cells

For the *in vitro* differentiation studies, B cells from spleen were purified from female eight-week-old mice. B cells were cultured in serum-free TexMacs medium (Miltenyi Biotec) and stimulated with 2.5 µg/mL anti-IgM and 2.5 µg/mL anti-CD40 antibodies, with or without PRL (50 ng/mL). The culture was incubated for 48 h for the analysis of B-GCs and five days for ASCs at 37°C and 5% CO_2_.

### Cell staining of germinal center B cells, survival, and antibody-secreting cells

Cells differentiated *in vitro* and primary cells isolated from mice with different treatments were stained with Ghost Red to analyze the percentage of living cells (Ghost Red neg) for 20 minutes at room temperature. Then the cells were stained with anti-GL7 and anti-CD19 antibodies (to identify B-GCs) and anti-CD19 and anti-CD138 antibodies (to identify ASCs) for 20 minutes at 4°C in the dark with FACS buffer. To measure cell activation, cells were incubated with anti-CD80 and anti-CD86 antibodies after 24 h of differentiation.

To stain BCL6, Ki67, and IRF4, the cells were fixed with 2% paraformaldehyde (Sigma−Aldrich) and permeabilized using Transcription Factor Staining Buffer (eBioscience, San Diego) for 2 h at 4^0^C, the cells were washed and staining with the antibodies. The samples were read using a MACSQuant Analyzer 10 flow cytometer (Miltenyi Biotec) and data were analyzed with FlowJo 10 software (Tree Star, Ashland, OR USA). In all flow cytometry analyses, cell viability was assessed using the Ghost Red marker.

### Nb2 cell bioassay and determination of proliferation by CFSE

The rat lymphoma cell line (Nb2) was used to measure the PRL bioactivity as described by Tanaka et al. (23). Nb2 cells are of a pre-T cell origin and their proliferation is dependent on mammalian lactogens, such as prolactin. Cells were maintained in RPMI with 10% of FBS (fetal bovine serum, HyClone, Logan, UT, USA), 1% antibiotics (Invitrogen, Carlsbad, CA, USA), 1% sodium pyruvate (HyClone), and 0.1% β-mercaptoethanol (Invitrogen) at 37°C and 5% CO_2_. Subsequently, the cells were washed and incubated with RPMI with 1% FBS for 24 h to synchronize the cell cycle. Increasing concentrations of recombinant mouse PRL in Tex Macs medium were added to Cell cycle resumption. The proliferation of Nb2 cells was analyzed with the CellTrace CFSE kit (carboxyfluorescein succinimidyl ester, Invitrogen).

PRL-like bioactivity was assessed with supernatants of B-GCs differentiated *in vitro* for 48 h with anti-IgM antibody (2.5, 5 or 10 µg/mL) plus anti-CD40 (2.5 µg/mL) antibody. We included controls without PRL. In these controls, cells were treated with medium or medium plus monoclonal antibody against PRL to block the PRL present in the supernatants.

Four million cells (B cells or Nb2 cells) were washed with PBS-BSA 0.1%, centrifuged, and stained with CFSE for 14 minutes at 37°C. Cells were washed three times with PBS-BSA to stop the reaction and placed on ice for 5 minutes.

B cells were counted and incubated for 48 h with different stimuli to favor formation of B-GC cells (anti-CD40, 2.5 µg/mL, and anti-IgM, 2.5 µg/mL, antibodies with or without 50 ng/mL of PRL). The proliferation index was obtained using the plugins from FlowJo “Proliferation Tool” Nb2 cells were incubated for 72 h with supernatants of B-GCs induced cells, as described above, the supernatants were pre-incubated with anti-PRL antibody. The samples were read using a MACSQuant Analyzer 10 flow cytometer and the data were analyzed with FlowJo 10 software.

### Immunofluorescence staining of cells

*In vitro* differentiated B-GC cells with 2.5, 5, or 10 µg/mL IgM and CD40 (2.5 µg/mL) were used. Cells were placed directly onto loaded glass slides, fixed, and permeabilized with BD Cytofix/Cytoperm (BD Biosciences). Cells were incubated overnight with the following antibodies: biotinylated anti-mouse prolactin antibody from R&D SYSTEMS (Minneapolis, MN, USA) and anti-CD19 FITC (clone 6D5) from Miltenyi Biotec (Bergisch Gladbach, Germany). Subsequently, slides were incubated with the secondary antibody anti-rat IgG-AF488 (Jackson ImmunoResearch) and streptavidin APC (eBioscience) for 2 h. The slides were incubated with Hoechst (Invitrogen) for 10 minutes to stain the nuclei and were mounted with Vectashield (Vector Laboratories, CA, USA). Images were taken with a Nikon Ti Eclipse inverted confocal microscope (Nikon, ME, NY, USA) using NIS Elements v.4.50. Imaging was performed using a 20x objective lens (dry, NA 0.8). Images were analyzed using the FIJI ImageJ software.

### IL-6 concentration determination

IL-6 concentration determination was performed on the sera of experimental mice and on the supernatants of differentiated B-GCs at 48 h using the commercial assay The BD™ CBA Mouse IL-6 Flex Set (Cytometric Bead Array, BD Biosciences, cat. 5622236). The samples were read using a MACSQuant Analyzer 10 flow cytometer and data were analyzed with FlowJo 10 software.

### IL-6 secreting cells

B-GCs were differentiated *in vitro* as described above and then incubated for 5 h with Cell Stimulation Cocktail (cocktail of phorbol 12-myristate 13-acetate (PMA) and ionomycin) and Protein Transport Inhibitor Cocktail (cocktail of Brefeldin A and Monensin) (eBioscience). The cells were washed, incubated with Ghost Red and with anti-GL7-Pacific Blue and anti-CD19-FITC antibodies at 4°C 20 minutes, followed by washing, fixing and permeabilizing with BD Cytofix/Cytoperm. The cells were stained with an anti-IL-6 PE antibody (clone MP5-20F3). The samples were read using a MACSQuant Analyzer 10 flow cytometer and data were analyzed with FlowJo 10 software.

### Quantification of IgG1, IgG2b, IgG3, and IgM antibodies in supernatants and serum

Antibody concentrations were determined by ELISA. A MaxiSorp plate (96-well) was sensitized with F(ab)_2_ anti-IgM (Jackson ImmunoResearch Laboratories, Inc. West Grove, PA, USA) at a concentration of 5 µg/mL for IgM and with F(ab)_2_ anti-IgG (Jackson ImmunoResearch Laboratories) for IgG1, IgG2b, and IgG3 at a concentration of 2.5 µg/mL in bicarbonate buffer overnight at 4°C. We used BSA-5% to block the plated. After washed, the plate was incubated with the IgM, IgG1, IgG2b, or IgG3 standards (Invitrogen, Carlsbad, CA, USA) at different concentrations (0 ng/mL, 15.6 ng/mL, 31.2 ng/mL, 62.5 ng/mL, 125 ng/mL, and 250 ng/mL) and the cell culture supernatants from cells differentiated for 5 days with or without PRL, or serum from mice with or without hyper-prolactinemia were added. The plate was incubated and washed; the conjugate and the specific substrates for each isotype were added. Plates were read at 405 or 450 nm using an ELISA reader.

### RT−PCR for PRL receptor isoforms

TRIzol reagent (Invitrogen, Carlsbad, CA, USA) was used to extract RNA from *in vitro* differentiated B-GCs or from *in vivo* B-GCs, according to the manufacturer’s protocol. cDNA was generated using ProtoScript II Reverse Transcriptase (New England BioLabs, MA, USA). Subsequently, for the expression of the different isoforms of PRL receptor, real-time PCR was performed using the following primers synthesized by Integrated DNA Technologies (IDT, Coralville, IA, USA). As constitutive gene: β-actin 5′-GAGGAGGCTCTGGTTCAACA- 3′ (reverse) and 5′-CAGTAAATGCCACGAACGAA-3′ (forward) was used. To determine the PRL receptor isoforms, the primers used were: long isoform 5′-GCTTGAGGAATCAGCCAAGA-3′ (reverse) and 5′-AAAGCTGGCCAGATCTTTCTC-3′ (forward) and short isoform 5′-GAGCACAAGAACAGGGGAAA-3′ (reverse) and 5′-GGATGGTTTCACAATCCACA-3′ (forward). The samples were analyzed in a LightCycler II thermocycler (Roche, Germany) using FastStart Essential DNA Green Master (Roche). With the following conditions: incubation at 95°C for 10 minutes and 40 cycles at 95°C for 10 s, 60°C for 10 s, and 72°C for 8 s. Relative expression was determined using the 2^-ΔΔCt^ formula. As a positive control of the expression of the short and the long isoforms of the PRL receptor, the 4T1 murine breast cancer cell line was used.

### Signaling pathway analysis

Differentiated B-GCs without PRL were allowed to settle for 8 h in basal medium. Subsequently, the cells were incubated with PRL for 30 minutes. The cells were fixed with 1x BD Phosflow Lyse/Fix Buffer 5x (BD Biosciences)/10 minutes, with BD Phosflow Perm Buffer III (BD Biosciences); the cells were permeabilized to determine intracellular STAT1 (anti-pSTAT1 PE, clone A15158B, BioLegend), STAT3 (anti-pSTAT3 PE, clone 13A3-1, BioLegend), and STAT5 (anti-pSTAT5 PE, clone SRBCZX, eBioscience) phosphorylation. To determine AKT (anti-pAKT PE, clone REA359, Miltenyi Biotec) and ERK1/2 (anti-pERK1/2 PE, clone 6B8B69, BioLegend) phosphorylation, cells were treated with IC Fixation Buffer (eBioscience) for 30 minutes at 4°C, washed with FACS buffer or Perm/Wash (BD Biosciences), and incubated with the antibodies for flow cytometric analysis for 30 minutes at 4°C. The samples were read using a MACSQuant Analyzer 10 flow cytometer and the data were analyzed with FlowJo 10 software.

### Statistical analysis

The Shapiro−Wilks normality test was used to determine the distribution of the data, finding that the data presented a normal distribution (p>0.05). The results were analyzed according to the data distribution (mean and standard deviation). Dependent variables were compared using the paired Student’s t test. We use one-way ANOVA and Tukey test as *post-hoc* when we compare three or more groups. Results were considered statistically significant when p<0.05 (*p<0.05, **p<0.01, ***p<0.001). Statistical analysis of the data was performed with SPSS Statistics 27 software (IBM, Armonk, NY, USA).

## Results

### High levels of prolactin increase anti-dsDNA antibodies of the IgG3 isotype

**Figure 1:**
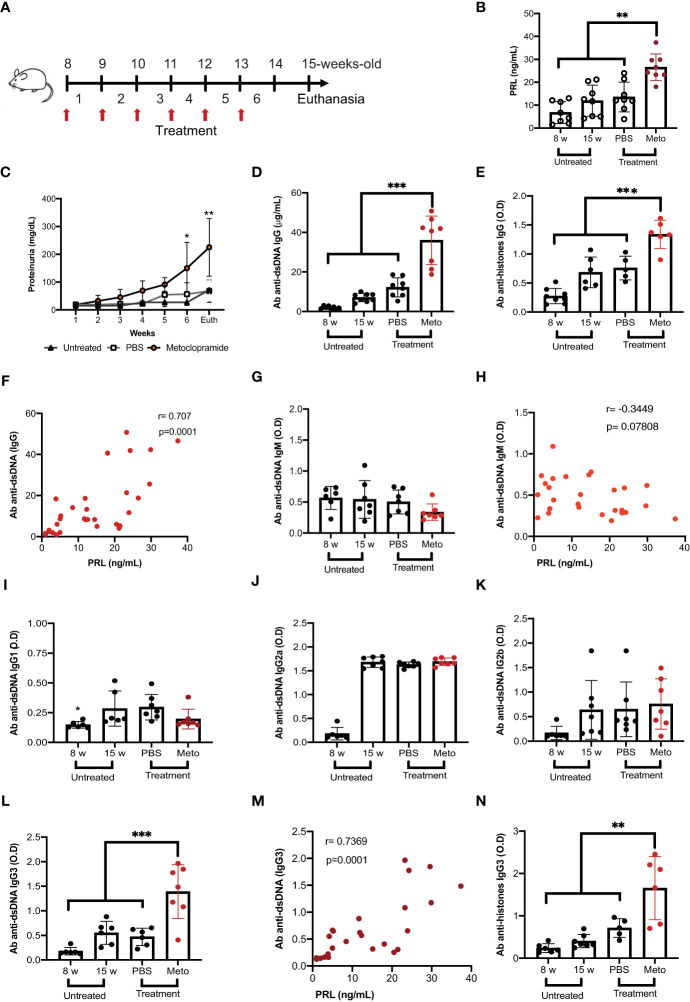
Prolactin correlates with increased levels of anti-dsDNA antibodies of the IgG3 isotype. Eight-week-old female MRL/lpr mice were treated subcutaneously with metoclopramide (meto, 200 µg) in 100 µL of PBS at five doses per week for six weeks. The control group was age and sex-paired mice that received 100 μL of PBS or no treatment during the same period. **(A)** Treatment strategy (flow chart). When the treatment was finished, the mice were sacrificed (Euth), after which the following parameters were measured. **(B)** The concentration of PRL by ELISA in mice with different treatments and in mice 8 weeks of age (statistical significant differences: Meto vs PBS p<0.01, Meto vs 15 weeks p<0.001, Meto vs 8 weeks p<0.001). **(C)** The concentration of proteinuria using reactive strips for urinalysis (determined every week during treatment). **(D)** The concentration of IgG anti-dsDNA antibodies by ELISA (Meto vs PBS p<0.001, Meto vs 15 weeks p<0.001, Meto vs 8 weeks p<0.001). **(E)** The concentration of IgG anti-histones antibodies by ELISA (Meto vs PBS p<0.001, Meto vs 15 weeks p<0.001, Meto vs 8 weeks p<0.001). **(F)** The correlation between the serum concentrations of anti-dsDNA IgG antibody and PRL. The optical density (O.D.) of the other isotypes of anti-dsDNA antibodies was also determined by ELISA. **(G)** IgM Isotype, **(H)** correlation between PRL and the O.D. of anti-dsDNA IgM, **(I)** IgG1, **(J)** IgG2a, **(K)** IgG2b, and **(L)** IgG3 (statistical significant differences: Meto vs PBS p<0.001, Meto vs 15 weeks p<0.001, Meto vs 8 weeks p<0.001). **(M)** Correlation between the concentrations of PRL and anti-dsDNA IgG3 antibody, and **(N)** IgG3 anti-histones (Meto vs PBS p<0.01, Meto vs 15 weeks p<0.001, Meto vs 8 weeks p<0.001). Eight mice were used per condition. Pooled data are presented as the mean ± SD. *p<0.05, ** p<0.01, ***p<0.001 using one-way ANOVA and Turkey´s *post-hoc* test. Correlations were obtained using Pearson’s correlation coefficient.

The concentration of PRL in serum correlates with the levels of autoantibodies and SLE clinical manifestations in patients (13). Our group reported that treating MRL/lpr mice with metoclopramide increases PRL serum levels (15, 16). Thus, we treated MRL/lpr and control mice with metoclopramide or PBS or left them untreated (**Figure 1A**, **Supplementary Figure 1**). We confirmed our previously published data that treatment with metoclopramide increased PRL levels (untreated mice at 8 weeks, 6.77 ± 4.46 ng/mL; untreated mice at 15 weeks, 11.93 ± 6.35 ng/mL; PBS-treated mice at 15 weeks, 14.94 ± 5.12 ng/mL; metoclopramide-treated mice at 15 weeks, 27.73 ± 5.07 ng/mL),(**Figure 1B**). We observed an increase in proteinuria and in the concentration of anti-dsDNA (p<0.001) and anti-histones (p<0.001) IgG autoantibodies in the MRL/lpr strain treated with metoclopramide (**Figures 1C-E**). Although metoclopramide increased PRL in nonautoimmune C57BL/6 mice, no evidence of elevation of other SLE markers was observed (**Supplementary Figures 1A, B**). We found a positive correlation between the concentrations of anti-dsDNA and anti-histones antibodies of the IgG isotype and the concentration of PRL (r=0.707, p=0.0001; r=0.782, p=0.0001) (**Figure 1F**, **Supplementary Figure 2A**). Interestingly, we did not observe differences between the mice in the optical densities (O.D.) of the anti-dsDNA and anti-histones antibodies of the IgM isotype (**Figure 1G**, **Supplementary Figure 2B**), and not correlation was found between PRL and IgM anti-dsDNA antibody (r=-0.3449, p=0.07808) (**Figure 1H**). When we determined the O.D. of the anti-dsDNA and anti-histones antibodies of the different IgG isotypes (IgG1, IgG2a, IgG2b, and IgG3), we found an increase in the O.D. only of the IgG3 isotype in mice with elevated PRL concentrations (treated with metoclopramide) (mice at 8 weeks, anti-dsDNA 0.18 ± 0.07, anti-histones 0.24 ± 0.09; mice at 15 weeks, anti-dsDNA 0.55 ± 0.21, anti-histones 0.40 ± 0.09; PBS anti-dsDNA 0.47 ± 0.16, anti-histones 0.71 ± 0.09; metoclopramide-treated mice, anti-dsDNA 1.39 ± 0.50 (p<0.001), anti-histone 1.77 ± 0.80 (p<0.001)) (**Figures 1I-N**, **Supplementary Figure 2C-F**). Again, these enhanced levels of IgG3 autoantibody correlated positively (r=0.7369, p=0.0001) with PRL concentrations (**Figure 1M**). We did not observe that metoclopramide treatment affected total immunoglobulin levels of IgM and IgG, only the concentration of the IgG3 increased (**Supplementary Figures 2G-L**). Thus, PRL increases IgG3, and this particular isotype seems responsible for DNA and histone self-reactivity and the potential for tissue damage.

### Elevated prolactin levels increase the absolute number of germinal center B cells and antibody-secreting cells

**Figure 2:**
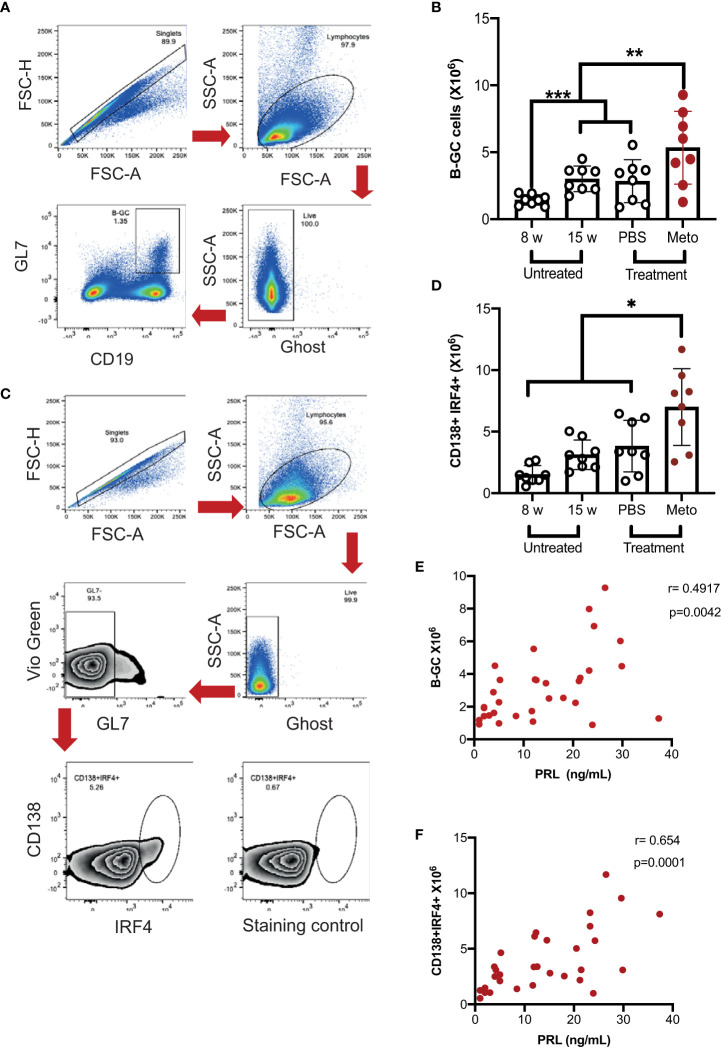
In MRL/lpr mice the increase in prolactin concentration correlates with the absolute number of germinal center B cells and antibody-secreting cells. Eight-week-old female MRL/lpr mice were treated subcutaneously with metoclopramide (meto) at five doses per week for six weeks, while the control group was age and sex-paired mice that received 100 μL of PBS or no treatment during the same period. At the end of the treatment, cells were stained with Ghost Red-APC-Cy7 (cell viability) and antibodies against CD19- PerCP-Cy5.5, GL7-Pacific Blue, BCL6-PE, IRF4-PE-Cy7 and CD138-PE. **(A)** Flow cytometry analysis strategy to gate on B-GC cells, FSC-H × FSC-A was used to excluded doublets; lymphocytes were gated on FSC-A × SSC-A plot and live cells were gated in the Ghost Red negative. The CD19+GL7+ cells represent the B-GCs. **(B)** Absolute number of B-GCs (statistical significant differences: Meto vs PBS p<0.05, Meto vs 15 weeks p<0.05, Meto vs 8 weeks p<0.001). **(C)** Flow cytometry analysis to gate on antibody-secreting cells (CD138+IRF4+). **(D)** Absolute number of antibody-secreting cells (CD138+IRF4+), (statistical significant differences: Meto vs PBS p<0.05, Meto vs 15 weeks p<0.01, Meto vs 8 weeks p<0.001). **(E)** Correlation between PRL concentrations and the absolute numbers of B-GCs. **(F)** Correlation between PRL concentrations and the absolute number of ASCs. Eight mice were used per condition. Pooled data are presented as the mean ± SD. * p < 0.05 using one-way ANOVA and Tukey´s *post-hoc* test. Correlations were obtained using Pearson’s correlation coefficient.

Since isotype switching occurs mainly in the GC, and PRL was found to correlate positively with anti-dsDNA IgG3 antibody, the absolute numbers of B-GCs (**Figures 2A, B**) and ASCs (CD138+IRF4+) (**Figures 2C, D**) were determined in mice (untreated, PBS-treated and metoclopramide-treated). We observed that the mice with the highest levels of PRL presented the highest absolute numbers of B-GCs (5.34 ± 2.72 X 10^6^ cells, p<0.05) and ASCs (7.00 ± 3.11 X 10^6^ cells, p<0.05). As expected, PBS-treated mice (B-GCs, 2.84 ± 1.60 X 10^6^; ASCs, 3.82 ± 2.10 X 10^6^ cells), and untreated 15-week-old mice (3.01 ± 0.95 X 10^6^; 3.12 ± 1.20 X 10^6^ cells) had significantly more B-GCs and ASCs than 8-week-old mice (1.43 ± 0.40 X 10^6^ cells, p<0.05; and 1.5 ± 0.73 X 10^6^ cells, p<0.05; respectively). The PRL serum levels correlated positively and significantly with the absolute numbers of B-GCs (r=0.49, p=0.004) and with ASCs (r=0.65, p=0.0001) (**Figures 2E, F**). We did not observe a metoclopramide/PRL effect on B-GCs and ASCs in control mice (C57BL/6) (**Supplementary Figures 1C, D**). These enhanced frequencies of B-GCs and ASCs induced by PRL are likely responsible for the high levels of self-reactive IgG3.

### Prolactin increases the proliferation of B-GCs

**Figure 3:**
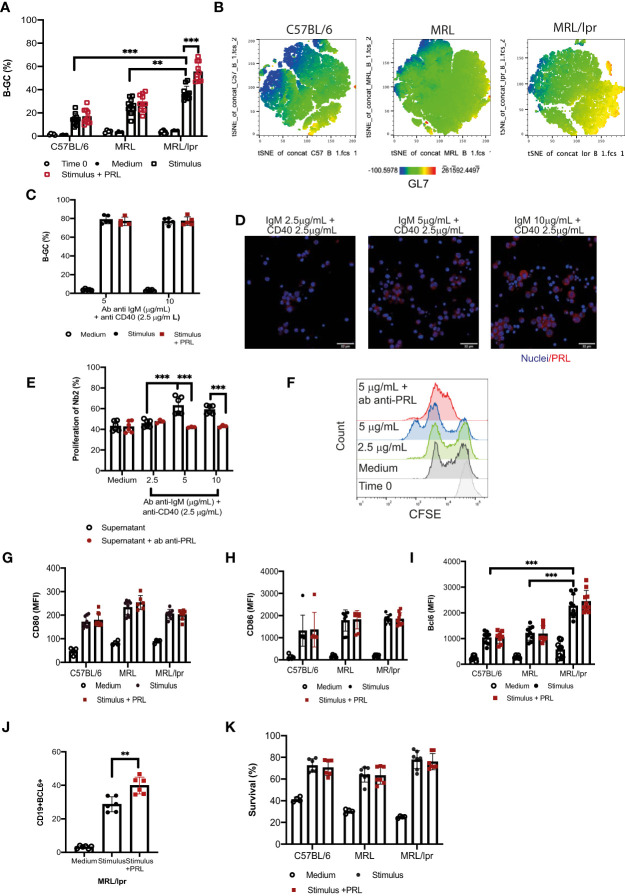
Prolactin increases the percentage of B-GCs in vitro. B-cell splenocytes from female 8-week-old C57BL/6, MRL, and MRL/lpr mice were purified by MACS cell separation (Time zero). The cells were differentiated into B-GCs with anti-CD40 (2.5 μg/mL) and anti-IgM (2.5 μg/mL) antibodies with or without PRL (50 ng/mL) for 48 h. B cells were stained with a viability marker (Ghost Red-APC-Cy7) and antibodies against CD19-PerCP-Cy5.5, GL7-Pacific-Blue and BCL6-PE. **(A)** Percentage of differentiation of B-GCs. **(B)** t-distributed stochastic neighbor embedding (t-SNE) plots for each mouse strain showing an enriched GL7-expressing population in the MRL/lpr mice. Eight independent experiments were performed. **(C)** Purified B cells from MRL/lpr mice were differentiated with anti-CD40 antibody (2.5 µg/mL) and with different concentrations of anti-IgM antibody (5 µg/mL and 10 µg/mL) with or without PRL (50 ng/mL) for 48 h. Five independent experiments were carried out, and each experiment was done in duplicate. **(D)** Immunofluorescence staining of PRL produced by B-GC cells. **(E)** The presence of PRL in the supernatants of B-GC cells was determined by measuring the proliferation of Nb2 cells. CFSE-labeled Nb2 cells were incubated with supernatants of B-GCs that were differentiated with anti-CD40 (2.5 µg/mL) and different concentrations of anti-IgM (2.5, 5, and 10 µg/mL), six independent experiments were carried out. The controls were medium alone or medium plus anti-PRL antibody. In the latter, supernatants were pre-incubated with anti-PRL antibody to block PRL activity, three independent experiments were carried out, and each experiment was done in duplicate. **(F)** Representative histograms of Nb2 cell proliferation. Purified B cells were incubated with anti-CD40 (2.5 μg/mL) and anti-IgM (2.5 μg/mL) antibodies with or without PRL (50 ng/mL) for 24 h, and the cells were stained with Ghost Red and antibodies against CD19-PerCP-Cy5, CD80-PE, and CD86-PE. Mean fluorescence intensity (MFI) of **(G)** CD80 and **(H)** CD86. Purified B cells were stimulated as in **(A)** and then stained with Ghost Red and antibodies against CD19, GL7, and BCL6. **(I)** MFI of BCL6 in B-GCs, **(J)** percentage of CD19+ BCL6+ cells (B-GCs) and **(K)** percentage of surviving B-GCs differentiated for 48 h in the presence and absence of PRL. Six to eight independent experiments were carried out. Pooled data are shown as the means ± standard deviations (SD). ** p < 0.01, *** p < 0.001 using one-way ANOVA and Tukey´s *post-hoc* test.

We determined whether PRL affects B-GC differentiation *in vitro* in MRL/lpr mice compared with C57BL/6 controls and included the MRL mouse strain, which is the parental strain for MRL/lpr. For this, we obtained splenic B cells from female 8-week-old mice in which there were no differences between the frequencies of developing B-GCs (**Figure 3A**). After purification, B cells were stimulated with anti-CD40 and anti-IgM antibodies and incubated with or without PRL (50 ng/mL). We found that the B cells from the MRL/lpr strain had a significant increase in the frequency of B-GCs (37.02 ± 4.89%) compared with the MRL (25.91 ± 6.65%; p<0.01) and C57BL/6 (14.61 ± 4.89; p<0.001) strains, which was also visualized by t-distributed stochastic neighbor-embedded (t-SNE) projections (**Figures 3A, B**) (24). Moreover, PRL further increased the percentage of B-GCs only in the MRL/lpr strain (without PRL, 37.02 ± 4.89%; with PRL, 55.41 ± 8.20%; p=0.001) (**Figure 3A**). This B-GC percentage increase was only observed when the MRL/lpr mouse B cells were incubated with suboptimal concentrations of anti-IgM (2.5 μg/mL) and anti-CD40 (2.5 μg/mL) antibodies (**Figure 3A**).

The effect of PRL was not observed at higher concentrations of anti-IgM antibodies (5 μg/mL and 10 μg/mL) (**Figure 3C**). This may be because B cells can secrete PRL, and this endogenous PRL could affect the effect of the exogenous PRL added to the cultures. Therefore, the presence of PRL in the B-GCs and in the supernatants of B cells incubated with a constant concentration of anti-CD40 antibody (2.5 μg/mL) and with different concentrations of anti-IgM antibody was determined. We observed that B-GCs produced PRL by immunofluorescence staining (**Figure 3D**). To confirm that B-GC cells are secreting PRL, the presence of PRL in the supernatants of B cells was determined by measuring the percentage of Nb2 cell proliferation (cells that need PRL to proliferate). We found that the supernatant of the B cells incubated with a constant concentration of anti-CD40 (2.5 μg/mL) and anti-IgM antibodies a different concentration (5 and 10 μg/mL), increased the proliferation of the Nb2 cells (63.00 ± 7.76%, p<0.001 and 59.03 ± 3.66%, p<0.01, respectively) compared with the supernatant of the cells incubated with the lowest anti-IgM tested (2.5 μg/mL; 45.58 ± 3.29%). As a control for this assay, we used medium (43.11 ± 4.23%) (**Figures 3E, F**). Also, the Nb2 proliferation decreased when the supernatant from B cells was pre-incubated with an antibody against PRL (IgM 5 μg/mL = 47.06 ± 1.38 p<0.001; IgM 10 μg/mL = 42.10 ± 0.40 p<0.001). These data suggest that PRL further exacerbates MRL/lpr mice, a strain already inclined to have an active B-GC differentiation pathway.

To establish the possible mechanism by which PRL increases the percentage of B-GCs, we determined the expression of the activation molecules CD80 and CD86 24 h after PRL stimulation. However, PRL did not affect the expression of these proteins (**Figures 3G, H**), suggesting that PRL does not increase B cell activation. We also determined whether PRL could promote differentiation into B-GCs by measuring the expression of BCL6, a transcription factor essential for the GC reaction and B-GC formation. We found that BCL6 expression was higher in the B-GCs of the mice that developed SLE compared with the control strains (p<0.001), further supporting that the B cells of the MRL/lpr strain are prone to form B-GCs (**Figure 3I**). Although, the expression of BCL6 measured through MFI did not increase (**Figure 3I**), the percentage of BCL6+ B-GC cells increased when the cells were differentiated with PRL (**Figure 3J**). Thus, we interpretate these data as PRL may not increase the B-GC cell differentiation, but that it may promote an alternative mechanism to increase the number of differentiating B-GCs. PRL can increase the survival of immature B cells in MRL/lpr mice (20). However, we did not observe an increased survival of PRL-treated B-GCs in the culture conditions (**Figure 3K**). Therefore, the PRL-mediated enhanced survival of differentiating B-GCs does not explain their increased frequency.

**Figure 4:**
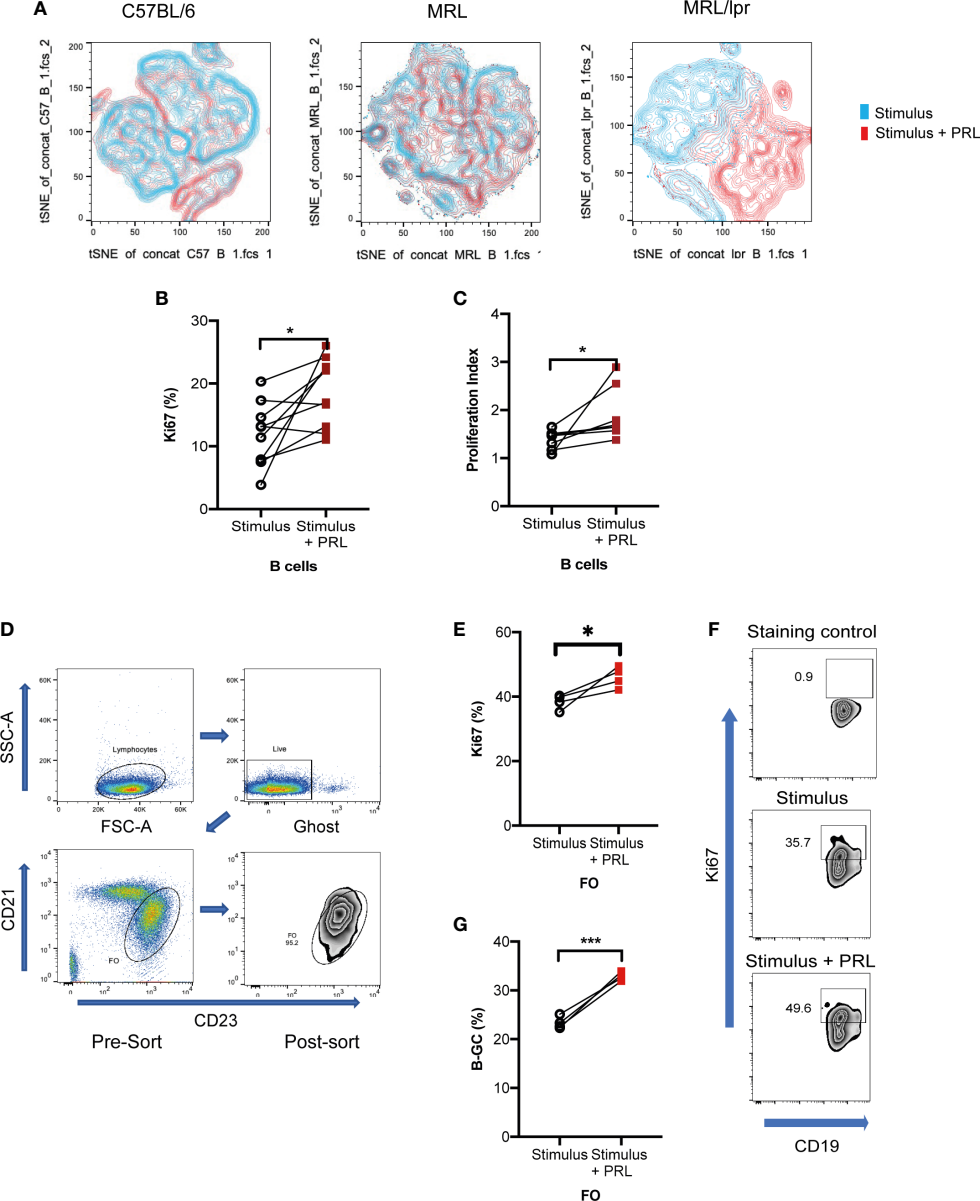
Prolactin increases B-GC cell proliferation. Purified B cells from 8-week-old female C57BL/6, MRL, and MRL/lpr mice were differentiated into B-GCs with or without PRL (50 ng/mL) for 48 h, and then stained with Ghost Red-APC-Cy7- and antibodies against CD19-PerCP-Cy5.5, GL7-Pacific Blue, BCL6-PE, Ki67-Alexa488, and IRF4- PE-Cy7. **(A)** t-SNE plots for each mouse strain. **(B)** Percentage of Ki67-expressing B-GCs in MRL/lpr mice. **(C)** B-GCs proliferation index. Eight independent experiments were performed, * p < 0.05. Follicular B cells (FO) from 8-week-old female MRL/lpr mice were sort-purified and differentiated to B-GCs with or without PRL (50 ng/mL). **(D)** Demonstration of the gating strategy to sort FO-B cells. **(E)** Percentage of Ki67 expression in B-GCs (from FO cells), **(F)** examples of zebra plots of Ki67+ cells, and **(G)** percentage of differentiated B-GCs (from FO cells). Three independent experiments were carried out, and each experiment was conducted in triplicate. * p < 0.05, *** p < 0.001 using a Student’s T test for paired samples.

The t-SNE visualization of B-GCs from the MRL/lpr strain shows that the cells were clustered into two distinct populations in the presence of PRL (**Figure 4A**). Since PRL favors an increase in the percentage of B-GCs without affecting their activation or differentiation into BCL6+ B-GCs, we determined whether PRL could affect their proliferation. We observed an increase in the percentage of Ki67^+^ B-GCs when the *in vitro* differentiation was carried out in the presence of PRL (without PRL, 11.70 ± 4.76%; with PRL, 18.68 ± 5.13%; p=0.0135) (**Figure 4B**). This was verified by carrying out proliferation assays with CFSE, the proliferation index increased when the B cells were differentiated with PRL (**Figure 4C**). The effect of PRL on proliferation was confirmed using purified FO B cells (instead of total B cell splenocytes) sorted from 8-week-old MRL/lpr mice (**Figure 4D**). We observed that PRL also favors the proliferation and formation of B-GCs when FO B cells are used (**Figures 4E-G**).

### Prolactin increases the concentration of IL-6 in B-GCs supernatants

**Figure 5:**
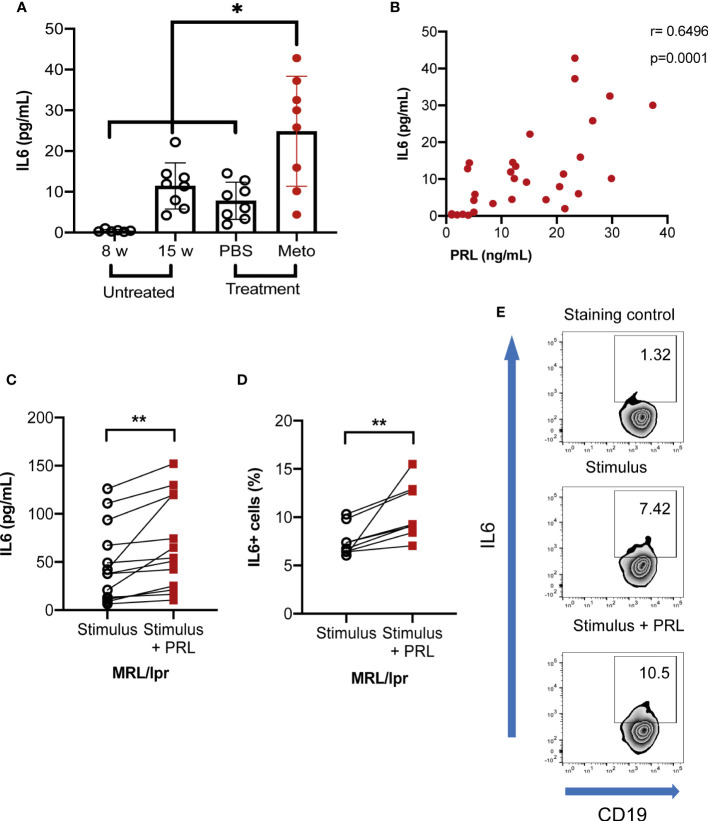
Prolactin increases the concentration of IL-6. Eight-week-old MRL/lpr female mice were left untreated or were treated with PBS or metoclopramide (meto) for six weeks (*in vivo*). **(A)** The IL-6 serum concentration was determined by a cytometric bead array (statistical significant differences: Meto vs PBS, p<0.01; Meto vs 15 weeks, p<0.05; Meto vs 8 weeks p<0.001). **(B)** Correlation between IL-6 and PRL concentrations. Eight mice per condition were used. Pooled data are presented as the mean ± SD. *p<0.05, using one-way ANOVA and Tukey´s *post-hoc* test. Correlations were obtained using Pearson’s correlation coefficient. Purified B cells from eight-week-old female MRL/lpr mice were differentiated into B-GCs with or without PRL (50 ng/mL) for 48 h, then incubated for five hours with a cell activation cocktail (plus protein transport inhibitors), and stained with Ghost Red and antibodies against CD19, GL7, and IL-6 (*in vitro*). **(C)** IL-6 concentration in B-GCs supernatants. **(D)** Percentage of IL-6+ B-GCs and **(E)** example of a zebra plot of IL-6 expression. Nine independent experiments were performed. **p< 0.01, using a Student’s T test for paired samples.

Although the GC is maintained by the expression of multiple cytokines, most of them are secreted by reticular or dendritic follicular cells. Of particular interest is IL-6, since it is also secreted by B-GCs (25). We assessed whether hyperprolactinemic MRL/lpr mice increased the secretion of IL-6 and found enhanced levels of IL-6 in metoclopramide-treated mice (p<0.05), and a positive correlation between the levels in serum of PRL and IL-6 (**Figures 5A, B**). Since these systemic IL-6 levels may be explained by multiple factors affecting the MLR/lpr mice, we also measured the concentration of IL-6 in the supernatants of the *in vitro* differentiated B-GCs in the presence and absence of PRL. We found that the IL-6 concentration and the percentage of IL-6+ cells increased when the cells were differentiated in the presence of PRL (**Figures 5C-E**), further supporting that PRL promotes IL-6 secretion in B-GCs in lupus-prone mice.

### Prolactin favors the differentiation of antibody-secreting cells and increases the concentration of the IgG3 isotype

**Figure 6:**
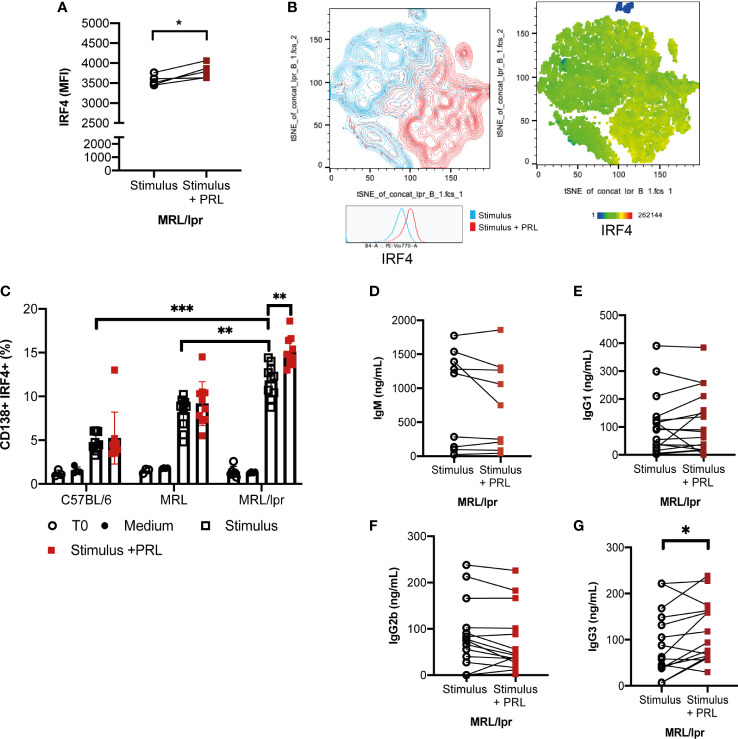
Prolactin promotes the development of IRF4+ antibody-secreting cells, and an enhanced concentration of IgG3 antibodies. Purified B cells from 8-week-old female MRL/lpr mice (Time zero) were differentiated into B-GCs with or without PRL (50 ng/mL) for 48 h and stained with Ghost Red and antibodies against CD19, GL7, BCL6, and IRF4. **(A)** MFI of IRF4. **(B)** t-distributed stochastic neighbor embedding (t-SNE) plots showing the population with enhanced expression of IRF4. Pooled data are presented as the mean ± SD *p< 0.05 using a Student’s T test for paired samples. B cells purified from 8-week-old female C57BL/6, MRL/lpr, and MRL mice were differentiated into ASCs with or without PRL (50ng/mL) for five days, and cells were stained with Ghost Red and antibodies against CD19, GL7, CD138, and IRF4. **(C)** Percentage of differentiation to ASCs (CD138+, IRF4+). Eight to nine independent experiments were performed. Pooled data are presented as the mean ± SD. **p < 0.01, ***p < 0.001 using one-way ANOVA and Tukey´s *post-hoc* test. In the supernatants of these cells, the concentrations of the isotypes **(D)** IgM, **(E)** IgG1, **(F)** IgG2b, and **(G)** IgG3 were determined by ELISA. *p< 0.05 using a Student’s T test for paired samples.

IRF4 is a critical transcription factor for initiating the differentiation of ASCs (26, 27). Our results showed that PRL increased IRF4 expression in B-GCs only in MRL/lpr mice (without PRL, 3560 ± 114.98 MFI; with PRL, 3798 ± 160.17 MFI, p<0.05), probably favoring differentiation to ASCs (**Figures 6A, B**). We determined the percentage of ASCs (CD138+IRF4+) after differentiating B cells for 5 days with or without PRL (50 ng/mL). The results showed that the B cells from the MRL/lpr strain had a higher potential for ASC differentiation (11.81 ± 1.77%) than the B cells from the MRL (8.19 ± 1.70%; p<0.05) and C57BL/6 strain (4.75 ± 0.98%; p=0.001). PRL further increased this potential to form ASCs in the MRL/lpr strain (15.10 ± 1.60%; p<0.01) (**Figure 6C**). The concentration of the different isotypes of antibody present in the supernatants was also determined, which confirmed that the IgG3 isotype concentrations increased in the supernatants of the MRL/lpr strain B cells that were differentiated with PRL (p<0.05) (**Figures 6D-G**). This was not observed in the other two strains (**Supplementary Figures 3, 4**).

### Prolactin signals through STAT1 on B-CG cells

**Figure 7:**
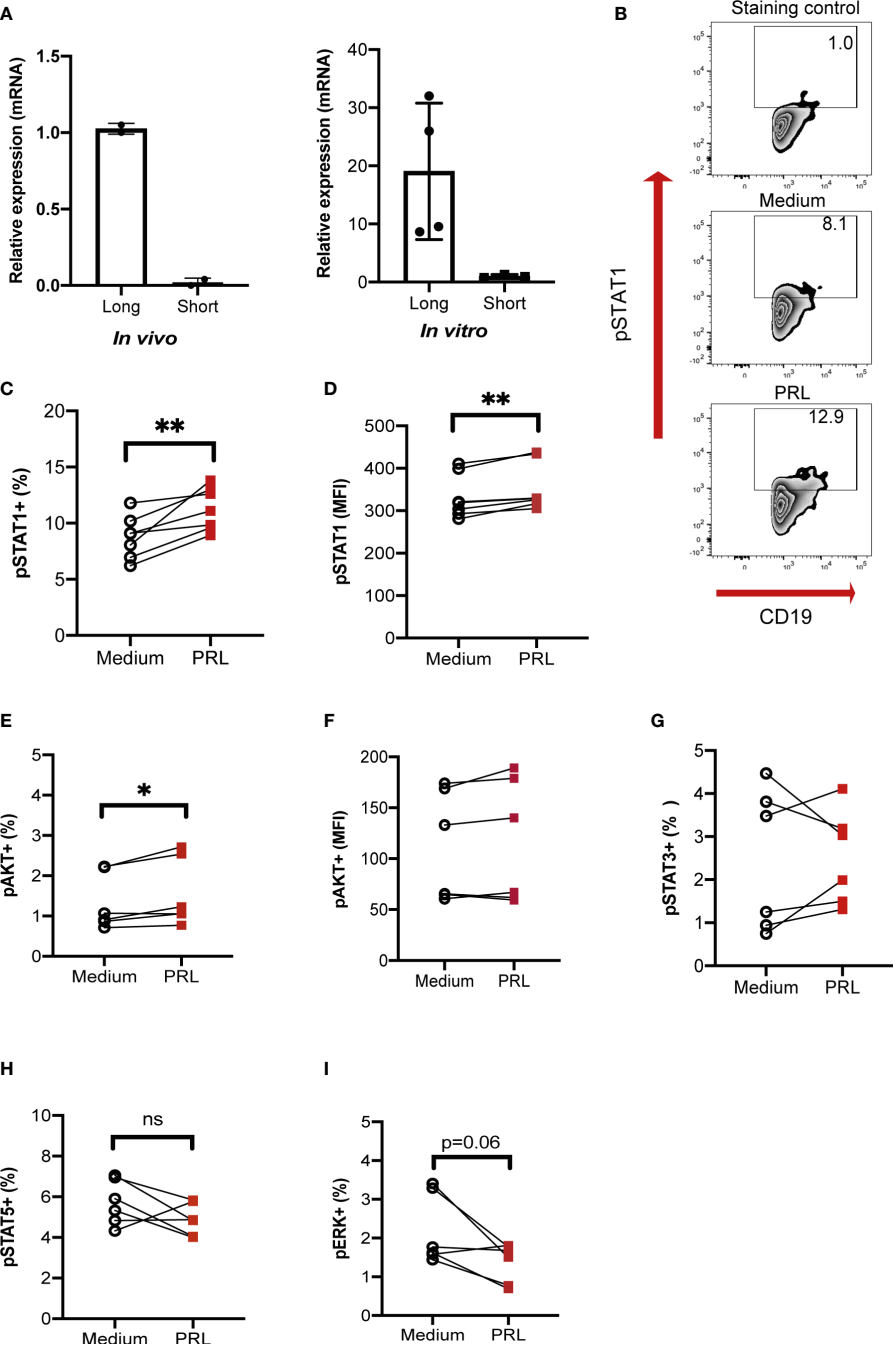
PRL signals through the long receptor isoform and STAT1 in B-GCs. B cells purified from female MRL/lpr mice were differentiated into B-GCs for 48 h (*in vitro*), and B-GC cells were obtained directly from 15-weeks-old female MRL/lpr mice (*in vivo*). **(A)** The relative expression and identity of the PRL receptor isoforms were determined by real-time (RT)-PCR (normalized to the endogenous gene β-actin and using the breast cancer cell line 4T1 as a control for the expression of both isoforms). To determine the percentage of phosphorylated cells, B-GCs were differentiated for 48 h, left in medium for 8 h and then stimulated for 30 min with PRL to determine **(B)** zebra plots of pSTAT1, **(C)** the percentage of pSTAT1, **(D)** MFI of pSTAT1 **(E)** the percentage of pAKT, **(F)** MFI of pAKT, and the percentages of **(G)** pSTAT3, **(H)** pSTAT5, and **(I)** pERK. Six independent experiments were performed. Pooled data are presented as the mean ± SD. *p < 0.05, **p < 0.01, ns, no statistical differences using a Student’s T test for paired samples.

**Figure 8:**
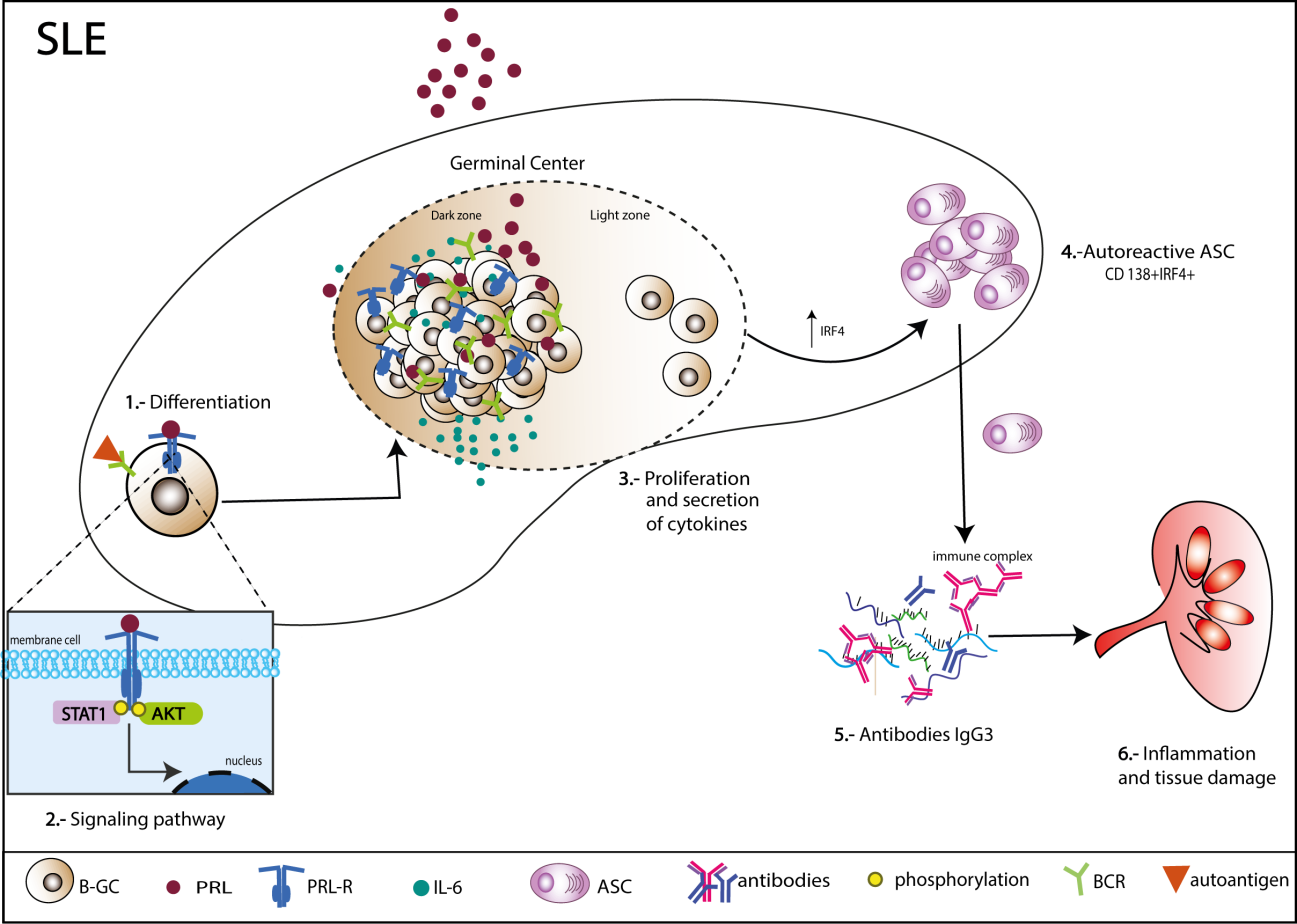
Mechanism of action of PRL in germinal center B cells (B-GCs) in SLE-susceptible mice. B-GCs from MRL/lpr mice that develop SLE express the long isoform of the PRL receptor. PRL can signal to a greater extent through STAT1 and increase the proliferation of B-GCs. Additionally, PRL can induce an increase in IRF4, which would favor ASC differentiation (CD138+IRF4+) and an increase in autoantibodies, especially of the IgG3 isotype, which could lead to the formation of immune complexes capable of depositing in multiple organs and causing tissue damage.

Finally, the pathway by which PRL signals in B-GCs was determined. There are different isoforms of the PRL receptor in mice (short and long isoforms), and the different isoforms signal through different pathways. We first determined the isoform of the PRL receptor expressed in B-GC cells from 15 weeks MRL/lpr mice (*in vivo*) and in *in vitro* differentiated B-GCs by real-time PCR. We used the breast cancer cell line 4T1 as a control for expression of both isoforms (**Supplementary Figure 5A**). We observed that mainly the long isoform was expressed in B-GC cells (**Figure 7A**). However, both isoforms were expressed in B-GC cells from C57BL/6 and MRL mice (**Supplementary Figure 5B**). Since STAT1, STAT3, STAT5, AKT, and ERK can be activated by the long isoform of the PRL receptor, the phosphorylation (p) of each of these proteins in B-GCs was determined by phospho flow cytometry. Our results demonstrated that PRL increased the percentage of pSTAT1+ B-GCs and the MFI of pSTAT1 in both the MRL/lpr (**Figures 7B-D**) and MRL mice (**Supplementary Figures 5C, D**). PRL also slightly increased the percentage of pAKT only in the MRL/lpr mice but without an increase in the MFI (**Figures 7E, F**, **Supplementary Figure 5**). PRL did not influence the percentage of positive cells (**Figures 7G-I**, **Supplementary Figure 5**) or the MFIs (not shown) of pSTAT3, pSTAT5, and pERK in B-GCs. Taken together, these data support that B-GCs from the SLE-prone MRL/lpr mouse strain express the long PRL receptor and that PRL signals through this receptor are mediated by pSTAT1 and perhaps pAKT. PRL signaling favors the expansion of these cells and the differentiation of significantly more autoreactive ASCs with the capacity to secrete larger numbers of IgG3 antibodies, some directed against autoantigens, such as DNA and histones, and with the potential to lead to tissue damage (see **Figure 8** for our current working model).

## Discussion

A positive correlation between PRL and anti-dsDNA antibody concentrations has been demonstrated in SLE patients (13), and women with hyperprolactinemia have a higher prevalence of autoantibodies (14). However, the molecular mechanism through which PRL increases autoantibody levels is unclear. Here, we observed a positive correlation between serum concentrations of PRL and anti-dsDNA autoantibodies of the IgG3 isotype (r=0.7369; p=0.0001), while IgM was negatively correlated (r=-0.3449; p=07808), suggesting a potential influence of PRL in class-switch recombination, a central process of the GC reaction (28). In the MRL/lpr and other murine models of autoimmunity, the presence of spontaneous GC has been demonstrated in the absence of detectable infection or immunization (2, 29). Different factors contribute to the development of spontaneous GC and enhanced risk of developing autoimmunity in humans and mice, such as BAFF (30, 31) and IFNγ−receptor (32, 33) overexpression, inflammatory cytokines, constitutive CD40 signaling (34), and human endogenous retrovirus (HERV) expression (35), etc. PRL could act as one of these factors, since our results also showed a positive correlation between PRL levels and absolute numbers of B-GCs in the spleen, and *in vitro* differentiation of FO B cells showed preferential formation of B-GCs.

It will be interesting to study whether other hormones that have been associated with SLE activity and whose secretion is also induced by metoclopramide, such as growth hormone (36, 37), and estrogens (38, 39) could also influence the formation of GC.

PRL does not seem to affect the formation of mature FO and MZ B cells, since we did not find differences in the absolute numbers of these populations (16). However, in T cells PRL increases the expression of CD25 and CD69 (activation molecules) on T cells (40, 41), and of OX40 on follicular T cells (42). We did not observe an effect of PRL on the expression of the B-cell activation markers CD80 and CD86. This is consistent with other studies in healthy subjects where B cells were incubated with PRL, and no effect on the expression of CD40 and CD86 was observed. Nevertheless, an increase in antibody production was reported (43). Therefore, PRL seems to differentially affect B cells and T cells.

PRL is a multifunctional hormone that is essential for the survival and proliferation of different cell types, both immune and nonimmune (44, 45). PRL regulates apoptosis by increasing the expression of antiapoptotic genes in both T cells (46), and immature B cells (20). Although in immature B cells from MRL/lpr mice, PRL can increase the survival and protect against apoptosis, it did not have this effect on already differentiated mature B cells (B-GCs), even though both cells express the long isoform of the receptor. This difference may be due to differential signaling pathway activation mediated by PRL at each stage of B-cell maturation, while in immature B cells, it seems to preferentially activate STAT3 (20). We observed here that it preferentially activates STAT1 in B-GC cells.

Interestingly, activation of STAT1 in B-GCs by PRL correlates with an increase in their proliferation, as measured by the number of Ki67+ B-GCs and an enhanced proliferation index (CFSE assay), suggesting that signaling through STAT3 favors survival of immature cells, while signaling through STAT1 favors proliferation of B-GCs. In agreement with these results, STAT3 is central to the transcriptional activation of the prosurvival genes *Bcl2a1a*, *Bcl2l2*, and *Birc5* (20). This is also supported by the report that IFNγ receptor (IFNγ-R) signaling controls spontaneous GC formation through phosphorylation of STAT1 and that proliferation and the percentage of B-GCs are affected in IFNγ-R^-/-^ mice (47). Furthermore, in MRL/lpr mice, the expression of STAT1 has been associated with increased kidney nephritis in MRL/lpr mice (48). Therefore, PRL could have a synergistic effect with the cytokines that phosphorylate STAT1, such as IFNγ, and that is important for GC formation (47). In future research, it will be important to determine the genes regulated by PRL and STAT1 signaling in B-GCs.

The increase in the proliferation of B-GCs by PRL also resulted in an increase in the percentage of IL6+ cells, and elevated concentrations of IL-6 in the serum of mice and supernatants of differentiated B-GCs correlated with high PRL concentrations. IL-6 serum levels are markedly increased in mice with autoimmunity (25, 49), and human patients with SLE (50, 51). IL-6 is required for the GC formation because it facilitates the expression of master regulator BCL6 in helper follicular T-cell differentiation. In B cells, the activation of STAT3 by IL6 is critical for initiating ASC differentiation programs (52, 53).

The generation of ASCs requires silencing of B-cell transcriptional programs and activation of a unique ASC transcriptome geared toward the production of large amounts of antibodies. This transition is achieved through the concerted function of the transcription factors XBP1, IRF4 and BLIMP1. We observed that PRL increased the levels of IRF4 in B-GCs. This increase, together with the increase in the concentration of IL-6 that activates the STAT3 pathway (27, 52, 54, 55), probably triggers ASC differentiation, leading to both an expansion in the absolute number of ASCs and an increase in the levels of autoantibodies.

The presence of autoantibodies against nuclear antigens, mainly against dsDNA, in the MRL/lpr strain has been extensively reported (56, 57). However, those studies only report the isotype (IgG). In the few studies in which IgG subclasses were determined, the main subclasses reported were IgG2a and IgG3 (58–62). We observed a remarkable specific correlation between PRL and anti-dsDNA and anti-histone antibodies of the IgG3 isotype. IgG3 protects mice against different bacterial infections (63). IgG3 is also the principal IgG isotype associated with kidney damage in mice. IgG3 causes glomerular injury independent of activation of complement. This capacity to induce damage may be due to other properties of IgG3. For instance, it has been shown that IgG3 has an ability to form non-covalent oligomers (64, 65), and this capacity of self-association seems to provide multivalent properties to IgG3 (66). For example, IgG3 induces hemagglutination with similar efficacy as IgM (67). In 2021 Alicja Karabasz et al, reported that an unknown IgG3-specific receptor is expressed on the surface of J774A.1 and P388D1 monocyte/macrophage-like cells (39). Activation of monocytes/macrophages through this receptor may contribute to Kidney damage. Furthermore, monoclonal antibodies of the IgG3 isotype directed against dsDNA were found to cause lesions similar to those described in human lupus nephritis in MRL/lpr (68) and (NZB×NZW)F1 (69). Hence, the IgG3 anti-dsDNA antibodies are an important factor in the development of glomerular lesions in the strains of mice that develop SLE (58, 70, 71). Thus, the kidney damage (proteinuria) repeatedly seen in mice with elevated PRL levels may be due to the increase in the pathogenic anti-DNA IgG3 autoantibodies. It is necessary to thoroughly dissect the specific mechanisms by which PRL facilitates the formation of IgG3-producing self-reactive ASCs to devise precision therapies.

## Limitations of the study

Among the weaknesses of the study is the lack of a more detailed B-GC analysis of the genes regulated by the PRL-activated pSTAT1 pathway that led to the preferential formation of IgG3-secreting autoreactive ASCs. We were also unable to block PRL receptor signaling *in vivo* to assess whether the lupus symptoms in the MRL/lpr mice would ameliorated.

## Data availability statement

The original data presented in the study are included in the article/**Supplementary Material**. Further inquiries can be directed to the corresponding author.

## Ethics statement

The animal study was reviewed and approved by Animal Care Committee of Instituto Nacional de Enfermedades Respiratorias (INER) “Ismael Cosío Villegas” and by the Hospital de Pediatría, Centro Médico Nacional Siglo XXI, IMSS (protocol numbers R-2016-785-050 and R-2021-3603-029).

## Author contributions

RC-T and PS-S worked to the design and performance of the experiments, analysis, and interpretation of the data. ML-H performed the experiments and analyzed the data. LC-S and PG-R contributed to the interpretation of data and statistical analyses. EF-P contributed to the design of experiments and supervised the editing and writing of the work. AC-R designed the experiments, supervised the experimental work, and wrote and edited the manuscript. All authors contributed to the article and read and approved the submitted version of the manuscript.

## Funding

This work was supported by CONACYT (grant number A1-S-9789).

## Acknowledgments

We want to express our appreciation the Flow Cytometry and Confocal microscopy core facility, the Coordinación de Investigación en Salud, CMN SXXI, IMSS, for the instrumental and technical support.

## Conflict of interest

The authors declare that the research was conducted in the absence of any commercial or financial relationships that could be construed as a potential conflict of interest.

## Publisher’s note

All claims expressed in this article are solely those of the authors and do not necessarily represent those of their affiliated organizations, or those of the publisher, the editors and the reviewers. Any product that may be evaluated in this article, or claim that may be made by its manufacturer, is not guaranteed or endorsed by the publisher.
