# Supplementary material for: Prolactin promotes proliferation of germinal center B cells, formation of plasma cells, and elevated levels of IgG3 anti-dsDNA autoantibodies
Source: Front Immunol. 2022 Oct 25;13:1017115. doi: 10.3389/fimmu.2022.1017115 (PMC9641231; doi:10.3389/fimmu.2022.1017115)
Supplement: Supplementary file 1 [file DataSheet_1.docx]

Supplementary Material.

## Supplementary Figures

(A)

(B)

(C)

(D)

**Supplementary Figure 1.** **Prolactin does not increase levels of anti-dsDNA antibodies, the absolute number of germinal center B cells, and antibody-secreting cells in C57BCL6 mice.** Eight-week-old female C57BCL6 mice were treated with metoclopramide (meto, 200 𝜇g/100 𝜇L), PBS (100 𝜇L), or without treatment (age) for 6 weeks. At the end of the treatment, it was determined: (A) the concentration of PRL by ELISA in mice with different treatments and in mice of eight weeks of age (statistical significant differences: Meto vs PBS p<0.001, Meto vs 15 weeks p<0.001, Meto vs 8 weeks p<0.001). (B) The concentration of auto-antibodies was determined in serum by ELISA, of the anti-dsDNA IgG isotype. (C) The absolute number of B-GC cells; (D) the absolute number of antibody-secreting cells (ASC, CD138+IRF4+). Six mice per condition were used. Pooled data are presented as mean ± SD. ** p<0.01 using one-way ANOVA and Tukey test as post-hoc.

(A)

(B)

(D)

(E)

(C)

(F)

(G)

(H)

(I)

(J)

(K)

(L)

**Supplementary Figure 2. Anti-histones antibodies, and concentration of different immunoglobulin in MRL/lpr mice.**

Eight-week-old female MRL/lpr mice were treated with metoclopramide (meto, 200 𝜇g/100 𝜇L), PBS (100 𝜇L) or were left without treatment (15 weeks) for 6 weeks. At the end of the treatment, the optical density (O.D.) of anti-histone antibodies was determined by ELISA. (A) The correlation between the O.D. of anti-histones IgG antibody and PRL was determined. Anti-histones antibodies IgM isotype (B), (C) IgG1, (D) IgG2a and (E) IgG2b. (F) Correlation between O.D. of anti-histones IgG3 antibody and PRL. The total immunoglobulin levels was determined by ELISA, (G) IgM, (H) IgG, (I) IgG1, (J) IgG2a, (K) IgG2b, and (L) IgG3. Pooled data are presented as mean ± SD. ** p<0.01 using one-way ANOVA and Tukey´s post-hoc test. Correlations were obtained using Pearson's correlation coefficient.

Count

**Supplementary Figure 3.** **T-distributed stochastic neighbor embedding (t-SNE) plots of the germinal center B cells in control mice.**

Purified B cells from female 8-week-old C57BL/6, and MRL mice were differentiated into B-GCs, in the presence or absence of PRL for 48 h, stained with a viability marker (Ghost Red) and antibodies anti-CD19, -GL7, -BCL6, -Ki67, and -IRF4. Histograms show no differences in the levels of these markers on C57BL/6 and MRL control mice.

(A)

(B)

(C)

(D)

**Supplementary Figure 4.** **The concentration of antibody subtypes in the supernatants of B cells derived from control mice.**

B cells purified from female 8-week-old C57BL/6 and MRL mice were differentiated into antibody-secreting cells in the presence or absence of PRL (50 ng/mL) for 5 days. in the supernatants of these cells, the concentration of antibodies of isotypes (A) IgM, (B) IgG1, (C) IgG2b and (D) IgG3 was determined by ELISA. *p< 0.05 using Student's T for paired samples.

(E)

(F)

(G)

(H)

(E)

(F)

(G)

(H)

(C)

(D)

(E)

(F)

(G)

(H)

(A)

(B)

**Supplementary Figure 5.** **Analysis of the signaling pathways activated by PRL in B-GC cells of control mice.**

(A) The relative expression and identity of the PRL-receptor isoforms were determined by real-time (RT)-PCR using the breast cancer cell line 4T1. (B) B cells purified from female C57BL/6 and MRL mice were differentiated into B-GC cells for 48 and the relative expression and identity of the PRL-receptor isoforms were determined by real-time (RT)-PCR. B cells purified from female C57BL/6 and MRL mice were differentiated into B-GC cells for 48 h left to rest for 8 h, and then incubated for 30 min with PRL to subsequently determine: (C) the percentage of pSTAT1+ cells, (D) MFI of pSTAT1; the percentage of (E) pSTAT3+ cells, (F) pSTAT5+ cells, (G) pAKT+ cells and (H) pERK+ cells. Six independent experiments were performed. Pooled data are presented as mean ± SD. *p < 0.05, using a Student's T test for paired samples. ns = non-significant.
